# Carbonic anhydrases from *Trypanosoma cruzi* and *Leishmania donovani chagasi* are inhibited by benzoxaboroles

**DOI:** 10.1080/14756366.2017.1414808

**Published:** 2017-12-27

**Authors:** Alessio Nocentini, Roberta Cadoni, Pascal Dumy, Claudiu T. Supuran, Jean-Yves Winum

**Affiliations:** a Institut des Biomolécules Max Mousseron (IBMM), UMR 5247 CNRS, ENSCM, Université de Montpellier, Montpellier Cedex, France;; b NEUROFARBA Department, Section of Pharmaceutical and Nutraceutical Sciences, University of Florence, Polo Scientifico, Firenze, Italy

**Keywords:** Benzoxaborole, *Trypanosoma cruzi*, *Leishmania donovani chagasi*, protozoan carbonic anhydrases, inhibition

## Abstract

A series of 6-substituted ureido- and thioureido-benzoxaboroles were investigated as inhibitors of carbonic anhydrases from *Trypanosoma cruzi* (TcCA), and *Leishmania donovani chagasi* (LdcCA). Both enzymes were inhibited by benzoxaboroles in the micromolar range. Preferential inhibitory potency against the β-CA LdcCA versus the α-CA TcCA was observed with submicromolar inhibitory activities. Some derivatives displayed excellent inhibitory and selectivity profile over the ubiquitous and physiological relevant human off-target hCA II. This study provides a convincing opportunity to study benzoxaborole scaffold for the design of antiprotozoan potential drugs targeting the pathogen’s carbonic anhydrases.

## Introduction

Chagas disease (American trypanosomiasis) and leishmaniasis belong to the list of neglected tropical diseases developed by the World Health Organization (WHO). Both of these diseases are caused by parasites belonging to the kinetoplastidae family and belong to the vector-borne diseases which are responsible for more than 17% of infectious diseases, affecting 20 million people and killing more than 50,000 every year^1^. Trypasonoma is transmitted by a variety of bedbugs, appeared in Latin America before spreading to other continents. In 30% of cases, it manifests itself in cardiac disorders, and, in 10% of cases, in digestive or neurological disorders. Leishmania, transmitted by the bite of an infected phlebotoma, causes skin or visceral ailments that are very debilitating or even fatal if left untreated. Current treatment used today has many limitations in terms of cost and toxicity, as well as the emergence of resistance phenomena throughout the world. Finding new therapeutic targets to develop new drugs is therefore urgent for these parasitoses, which WHO now classifies as priority infections (category 1: reemerging or uncontrolled infections)^2^^,^^3^.

The availability of the complete genome sequence of both protozoans has given the possibility of large-scale analysis, which lead to consequent identification of novel drug targets. Protozoan carbonic anhydrases (CAs, EC 4.2.1.1) were thus recently identified as novel promising targets for chemotherapeutic interventions^4–6^. Because of the universal reaction they catalyze, i.e. reversible hydration of CO_2_ to bicarbonate with a proton release, the prokaryotic metalloproteins are considered as important components in the growth and virulence of pathogenic microorganisms. In 2013, carbonic anhydrases from the two unicellular protozoans *Trypanosoma cruzi* (TcCA) and *Leishmania donovani chagasi* (LdcCA) were cloned and characterized^7^^,^^8^, paving the way for new opportunities in the design of novel inhibitors exploitable as antiprotozoal agents and acting by a totally new mechanism of action, lacking of cross-resistance to existing drugs.

Important classes of carbonic anhydrase inhibitors (CAIs) have been investigated in detail for their inhibitory profile against these two protozoans carbonic anhydrases.

The α-CA TcCA, which is characterized by very high catalytic activity for the CO_2_ hydration reaction (*k*_cat_ of 1.21 × 10^6^ s^−1^ and *k*_cat_/K_M_ of 1.49 × 10^8^ M^−1 ^s^−1^), was shown to be inhibited in the nanomolar range by many types of aromatic/heterocyclic sulfonamides^7^^,^^9^^,^^10^, sulfamates^7^, thiols^7^ and hydroxamates^11^; the two last families of inhibitors demonstrated anti-trypanosomal activity, inhibiting *in vivo* the three phases of the pathogen’s life cycle^5^^,^^7^^,^^11^. Inorganic anions and other small molecules were also reported to inhibit TcCA, with a simple dithiocarbamate showing low micromolar activity *in vitro*^5^^,^^12^.

The β-CA LdcCA which also possess an effective catalytic activity for the CO_2_ hydration reaction (*k*_cat_ of 9.35 × 10^5^ s^−1^ and *k*_cat_/*K*_M_ of 5.9 × 10^7^ M^−1 ^s^−1^), has been reported to be efficiently inhibited by sulfonamides and heterocyclic thiols with nanomolar inhibition constants^8^^,^^13^. Preliminary *in vivo* assays demonstrated that selected inhibitors in thiol series possessed anti-leishmania activity, being able to reduce parasites growth and causing their death^8^.

Considering the druggability of protozoans CAs, the design of new inhibitors with potent anti-trypasonomal or anti-leishmania activities is worth of investigation.

Previously, we reported a new generation of CAIs based on benzoxaborole scaffold. This new family of inhibitors were shown to act *via* a new binding mode, and to be effective against human α-CA as well as β-CA from pathogenic fungi^14^^,^^15^.

Encouraged by these results but also by the interesting profile of the trypanocidal orally active benzoxaborole compound SCYX-7158^16^^,^^17^ which enter Phase IIb/III trials in 2016 for the treatment of African trypanosomiasis, we report in this paper, the inhibitory activities of a series of 6-substituted urea/thiourea benzoxaboroles against CAs from the two pathogenic protozoans TcCA and LdcCA in order to detect possible candidates for anti-protozoans studies.

## Material and methods

### Chemistry

Compounds **1–23** were previously reported by this group^14^.

### CA inhibition assay

An applied photophysics stopped-flow instrument has been used for assaying the CA catalyzed CO_2_ hydration activity^18^. Phenol red (at a concentration of 0.2 mM) has been used as indicator, working at the absorbance maximum of 557 nm, with 20 mM Hepes (pH 7.5) as buffer for testing the α-CAs and with 20 mM TRIS buffer (pH 8.3) for testing the β-class enzyme. Na_2_SO_4_ of 20 mM were also added to the assay system for maintaining constant the ionic strength. The initial rates of the CA-catalyzed CO_2_ hydration reaction were followed for a period of 10–100 s. The CO_2_ concentrations ranged from 1.7 to 17 mM for the determination of the kinetic parameters and inhibition constants. For each inhibitor at least six traces of the initial 5–10% of the reaction have been used for determining the initial velocity. The uncatalyzed rates were determined in the same manner and subtracted from the total observed rates. Stock solutions of inhibitor (0.1 mM) were prepared in distilled-deionized water and dilutions up to 0.01 nM were done thereafter with the assay buffer. Inhibitor and enzyme solutions were preincubated together for 15 min at room temperature prior to assay, in order to allow for the formation of the E–I complex. The inhibition constants were obtained by non-linear least-squares methods using PRISM 3 and the Cheng–Prusoff equation, as reported earlier^19–23^ and represent the mean from at least three different determinations. All CA isoforms used in these experiments were recombinant ones obtained in-house as reported earlier^7^^,^^8^.

## Results and discussion

Inhibition data of benzoxaborole **1–23** against TcCA and LdcCA were measured by a stopped flow CO_2_ hydrase assay and are shown in Table 1. Acetazolamide, a clinically used sulfonamide inhibitor, was used as standard. SCYX-7158 being not commercially available, Tavaborole, a commercially benzoxaborole used as topical antifungal medication was also used as standard in our inhibition assays. Inhibitory activities were displayed in comparison with the ones against the two physiologically relevant and off-target human isoforms hCA I and hCA II (Table 1).

**Table 1.** Inhibition data of α-CA isoforms hCA I, hCA II, TcCA and β-CA isoform LdcCA, with benzoxaboroles **1–23**, tavaborole and the standard sulfonamide inhibitor acetazolamide by a stopped flow CO_2_ hydrase assay^15^. 
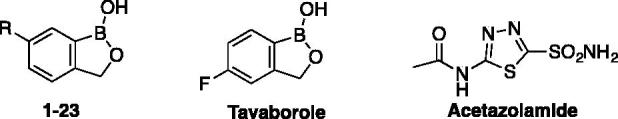

		*K_I_* (μM)^a^				Selectivity ratio		
Compounds	R=	TcCA	LdcCA	hCA I	hCA II	*K*_I_ (TcCA)/*K*_I_ (LdcCA)		*K*_I_ (hCA II)/*K*_I_ (LdcCA)
**1**	H	>100	3.79	5.69	8.18	>26		2.2
**2**	NO_2_	>100	4.01	6.35	0.50	>25		0.1
**3**	NH_2_	>100	2.37	9.43	0.60	>42		0.2
**4**	NHCONH-CH_2_Ph	75.1	2.04	0.56	0.44	36.8		0.2
**5**	NHCONH-CH_2_-(3-Cl,5-CH_3_-Ph)	87.6	4.30	0.56	0.28	20.4		0.1
**6**	NHCONH-Ph	32.3	0.74	0.65	0.73	43.6		1
**7**	NHCONH-(4-Cl-Ph)	37.8	0.62	3.46	0.71	61		1.1
**8**	NHCONH-CH_2_-fur-2-yl	67.6	3.54	0.61	0.84	19.1		0.2
**9**	NHCONH-(4-F-Ph)	24.0	0.78	0.23	0.48	30.8		0.6
**10**	NHCONH-(4-CF_3_-Ph)	60.7	2.59	0.49	0.46	23.4		0.2
**11**	NHCONH-(2,4,6-Cl-Ph)	69.5	3.85	0.45	0.27	18.1		0.1
**12**	NHCONH-(2-OMe,5-CH_3_-Ph)	38.6	0.67	0.10	0.09	57.6		0.1
**13**	NHCONH-(4-COCH_3_-Ph)	33.6	0.48	0.29	0.80	70		1.7
**14**	NHCSNH-CH_2_CH_2_Ph	72.8	3.20	0.64	1.55	22.7		0.5
**15**	NHCSNH-(4-CH_3_-Ph)	32.5	0.67	0.32	1.25	48.5		1.9
**16**	NHCSNH-napht-2-yl	58.8	3.06	0.55	1.15	19.2		0.4
**17**	NHCSNH-(4-OCH_3_-Ph)	12.6	0.59	0.51	1.25	21.3		2.1
**18**	NHCSNH-(4-NO_2_-Ph)	46.8	0.91	0.38	>100	51.4		110
**19**	NHCSNH-CH_2_Ph	59.6	4.37	0.38	1.30	13.6		0.3
**20**	NHCSNH-(4-F-Ph)	19.7	0.66	0.35	1.50	29.8		2.3
**21**	NHCSNH-CH_2_-fur-2-yl	42.1	4.36	0.26	2.23	9.6		0.5
**22**	NHCSNH-(4-CF_3_-Ph)	16.6	0.85	0.42	1.84	19.5		2.2
**23**	NHCSNH-Ph	23.4	0.65	0.53	1.62	36		2.5
Tavaborole		60.5	2.54	2.01	0.46	23.8		0.2
Acetazolamide		0.06	0.09	0.25	0.01	0.6		0.1

a Mean from three different assays, by a stopped flow technique (errors were in the range of ±5–10% of the reported values).

Regarding the data in Table 1, the following structure activity relationship for compounds **1–23** may be noted:

- Ureido and thioureido benzoxaborole **1–23** exhibited micromolar to low micromolar inhibitory activities against protozoans TcCA and LdcCA, showing a pronounced selectivity against the β-CA LdcCA with inhibition constant ranging from 3 to 0.65 μM, and selectivity ratio (*K*_I_(TcCA)/*K*_I_(LdcCA)) ranging from 9 to 70, the more selective being compound **13**. Against the TcCA isoform, *K*_I_s were ranging from 12.6 to 75.1 μM, the best inhibitor detected being the *para*-methoxy derivative **17**.
- It is worth noting that simple benzoxaborole **1**, 6-nitro-**2** and 6-amino benzoxaboroles **3** exhibited low micromolar activities against LdcCA, as the same level of magnitude as against hCA I, with inhibition constants ranging from 2.37 to 4.01 μM. No activity was noticed for these three compounds against TcCA (*K*_I_>100 μM). Tavaborole, a benzoxaborole used as standard in this study, showed inhibition potency similar to compound **2**, and **3**, against LdcCA, and a slight selectivity against human isoforms hCA II with a 5.5 magnitude selectivity ratio (*K*_I_(LdcCA)/*K*_I_(hCA II)).
- From a general point of view, change of the ureido by a thioureido group did not affect significantly inhibitory potency. Inhibitory activity against LdcCA was comparable with the ones observed for the off-targets hCA I and hCA II. A slight change of selectivity may be noticed for some compounds when we compare LdcCA versus hCA II. For example, if we compare inhibition data for the phenylureido benzoxaborole **6** and phenylthioureido benzoxaborole **23**, a selectivity of 2.5 magnitude for LdcCA was observed with the thioureido derivative **23** and no selectivity with the ureido compound **6**. Substitution in the *para* position of the phenyl ring (e.g. 4-F, **20** and 4-CF_3_, **22**) also showed a 2.2 magnitude of selectivity against LdcCA over CA II in thioureido series. An inversion of selectivity can be noticed for these two compounds in ureido series where compounds **9** (4-F derivative) and **10** (4-CF_3_) were, respectively, more selective against hCA II over LdcCA with selectivity ratio of 1.6 and 5.6 (*K*_I_(LdcCA)/*K*_I_(hCA II)).
- The most performing inhibitor was found to be the *para*-nitrophenyl thioureido derivative **18** with a high-selectivity ratio against the pathogenic isoform LdcCA over hCA II (around 110). This compound could represent a slightly better solution compared with the standard clinically used sulfonamide acetazolamide, as acetazolamide showed selectivity for hCA II over LdcCA and TcCA with selectivity ratio, respectively, of **6** and **9**.

## Conclusion

We report for the first time the activity of benzoxaborole derivatives against protozoans CAs. 6-Substituted ureido and thioureido benzoxaborole derivatives **4–23** investigated here showed a preferential inhibitory activity against the β-CA from *Leishmania donovani chagasi* (LdcCA) versus the α-CA from TcCA. Some derivatives such as **18** displayed excellent inhibitory and selectivity profile for LdcCA over the human off-target hCA II.

The present study demonstrates that benzoxaborole chemotype offers interesting opportunities for the inhibition of CA from pathogenic protozoans and for the development of anti-trypasonomal or anti-leishmania compounds with a new mechanism of action, warranting further development.
